# HCV^+^ Hepatocytes Induce Human Regulatory CD4^+^ T Cells through the Production of TGF-β

**DOI:** 10.1371/journal.pone.0012154

**Published:** 2010-08-13

**Authors:** Caroline H. T. Hall, Rachel Kassel, Robert S. Tacke, Young S. Hahn

**Affiliations:** 1 Department of Microbiology, Beirne B. Carter Center for Immunology Research, University of Virginia, Charlottesville, Virginia, United States of America; 2 Department of Pathology, University of Virginia, Charlottesville, Virginia, United States of America; New York University, United States of America

## Abstract

**Background:**

Hepatitis C Virus (HCV) is remarkably efficient at establishing persistent infection and is associated with the development of chronic liver disease. Impaired T cell responses facilitate and maintain persistent HCV infection. Importantly, CD4^+^ regulatory T cells (Tregs) act by dampening antiviral T cell responses in HCV infection. The mechanism for induction and/or expansion of Tregs in HCV is unknown.

**Methodology/Principal Findings:**

HCV-expressing hepatocytes were used to determine if hepatocytes are able to induce Tregs. The infected liver environment was modeled by establishing the co-culture of the human hepatoma cell line, Huh7.5, containing the full-length genome of HCV genotype 1a (Huh7.5-FL) with activated CD4^+^ T cells. The production of IFN-γ was diminished following co-culture with Huh7.5-FL as compared to controls. Notably, CD4^+^ T cells in contact with Huh7.5-FL expressed an increased level of the Treg markers, CD25, Foxp3, CTLA-4 and LAP, and were able to suppress the proliferation of effector T cells. Importantly, HCV^+^ hepatocytes upregulated the production of TGF-β and blockade of TGF-β abrogated Treg phenotype and function.

**Conclusions/Significance:**

These results demonstrate that HCV infected hepatocytes are capable of directly inducing Tregs development and may contribute to impaired host T cell responses.

## Introduction

Hepatitis C Virus (HCV) is problematic for worldwide human health, resulting in the development of chronic liver disease and liver cancer. HCV is highly efficient at establishing persistent infection, as 70–80% of infected individuals develop chronic HCV infection. Impaired antiviral CD8^+^ T cell and lack of CD4^+^ Th1 responses are associated with the persistence of HCV infection [1]. Although the failure of CD8^+^ T cell responses might occur as a result of mutation [2], [3] and the upregulation of negative costimulatory PD-1 and CTLA-4 pathways [4], [5], little is known about how HCV infection leads to inhibition of CD4^+^ T cell responses. Clinical studies suggest that CD4^+^CD25^+^FoxP3^+^ regulatory T cells (Tregs), cells known to maintain immune homeostasis and control excessive immune responses, participate in suppressing anti-viral T cell immunity against HCV infection. Indeed, an increase in the number and functionality of Tregs has been detected in chronic HCV patients as compared to those whose infection resolve [6], [7] [8].

The increased frequency of Tregs observed in chronic HCV patients might arise from the expansion of thymic-derived natural Tregs or from the *de novo* induction from naïve T cells. The mechanism underlying induction of Tregs during HCV infection remains undefined. The immunoregulatory cytokines, TGF-β and IL-10, are crucial for induction and maintenance of Tregs: TGF-β is involved in the generation of inducible Tregs and maintenance of Treg function [9], [10] and IL-10 is a critical factor for sustaining FoxP3 expression [11]. In addition, the production of these cytokines have been reported to be elevated during HCV infection, play a critical role in impairing HCV-specific T cell responses and have polymorphisms that correlate with HCV clearance[12]. Intracellular expression of HCV core has been demonstrated to enhance TGF-β mRNA production by the hepatoma cell line HepG2 [13] [14]. Additionally, a recent paper has identified an HCV-dependent increase in TGF-β that may be due to the production of reactive oxygen species[15]. However, another study found that HCV core expression within hepatoma cells resulted in a reduction in TGF-β promotor activity[16]. Therefore, the analysis of cytokine production by hepatocytes expressing the complete HCV genome and their immune modulatory function will be helpful to elucidate the regulation of host immune responses by HCV.

The primary site of HCV viral replication is within hepatocytes. Lymphocytes and hepatocytes have ample opportunity to contact one another due to the fenestrated structure of hepatic sinusoids, combined with the lack of basal membrane and the low velocity blood flow [17]. Although hepatocytes are not traditionally regarded as key players in the immune response, recent studies highlight the role of hepatocytes in the regulation of host immunity by soluble factors. Huh7 cells and primary hepatocytes are capable of producing lymphocyte regulating cytokines and chemokines such as IL-7, IL-15, TGF-β, TNF-α, IL-1β, RANTES, MIP-1α and IL-8 [18], [19], [20]. Although HCV proteins mainly remain within the hepatocyte, they may be able to modulate lymphocytic activity by the alteration of expression of these cytokines.

In this report, we examined whether HCV protein expression within hepatocytes alters the function of CD4^+^ T cells and could contribute to the development of Tregs. By using an HCV expressing hepatoma line, Huh7.5-FL, we evaluated the contribution of infected hepatocytes on CD4^+^ T cell dysfunction. CD4^+^ T cell responsiveness, as measured by IFN-γ production, was diminished in co-culture with Huh7.5-FL compared to controls. Importantly, CD4^+^ T cells in contact with Huh7.5-FL adopted a Treg phenotype (CD25^+^FoxP3^+^CTLA-4^+^LAP^+^) and developed the ability to suppress effector T cell proliferation. The role of hepatocytes in Treg development was clarified by the finding that Huh7.5-FL produced more TGF-β than control hepatocytes. Further, blockade of TGF-β production impaired the development of Tregs. These results suggest that the site of HCV infection (i.e. hepatocytes) plays a pivotal role in impairing the antiviral T cell response by the induction of Tregs.

## Results

### HCV^+^ hepatocytes inhibit IFN-γ production by CD4^+^ T cells

**Figure 1:**
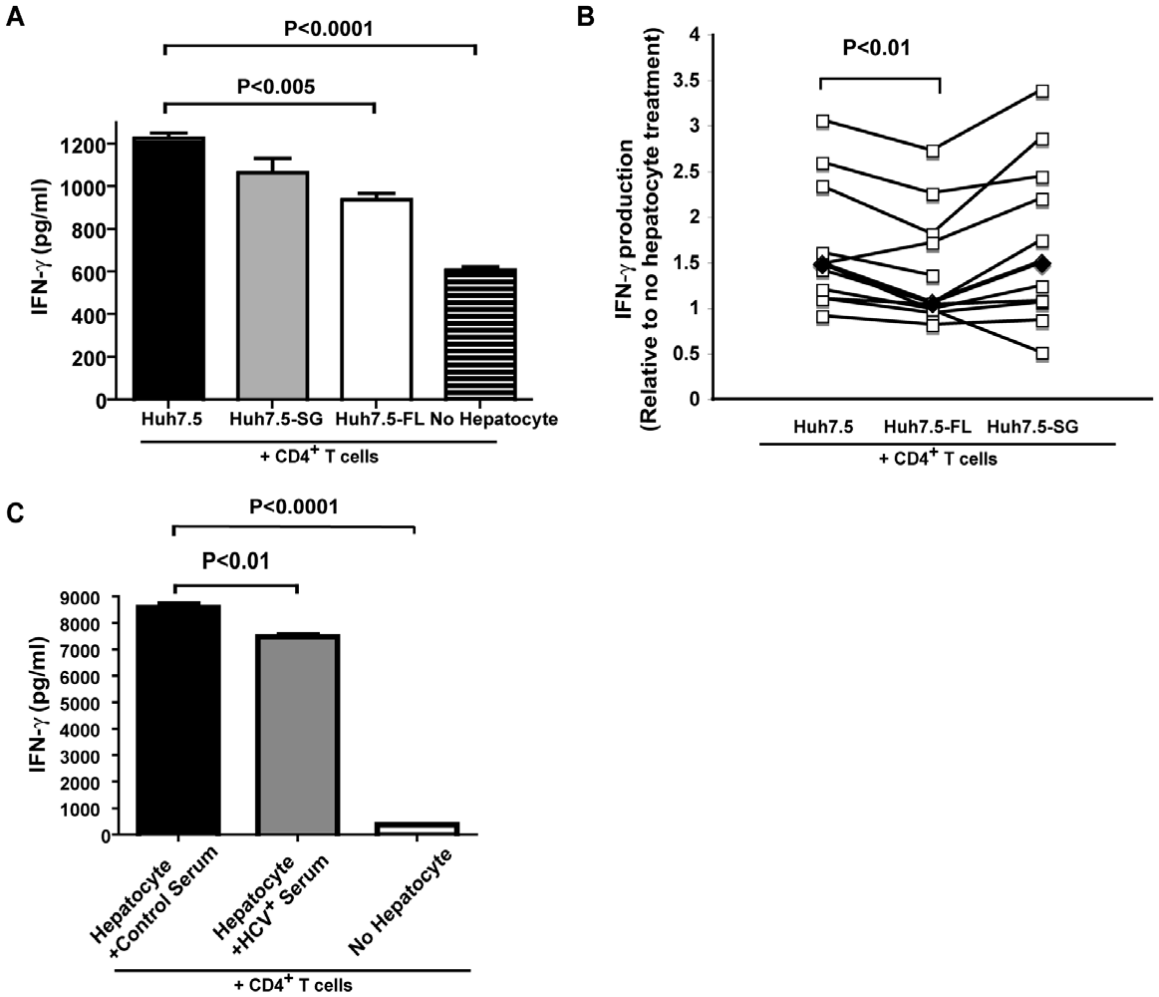
HCV^+^ hepatocytes decrease IFN-γ production by CD4^+^ T cells. A, B) Plate-bound anti-CD3/CD28 activated CD4^+^ T cells were co-cultured with Huh7.5 cells, as well as Huh7.5 cells stably transfected with the non-structural portion of the HCV genome (Huh7.5-SG) or the full HCV genome (Huh7.5-FL). The cells were cultured for 48 hrs in media containing 10 U/ml rhIL-2 at a ratio of 1∶1. The supernatant was analyzed by ELISA. IFN-γ production is presented relative to a no hepatocyte control. Data is representative of 10 healthy CD4^+^ T cell donors examined. C) Primary hepatocytes were cultured on matrigel and exposed to control serum or serum from genotype 1 HCV^+^ patients for 24 hrs. Following infection, cells were washed with media and allowed to proceed with infection for 5 days. RT-PCR analysis of HCV genome was performed to ensure infection had occurred. 5 days after infection, activated CD4^+^ T cells were added to the hepatocyte culture. Supernatant was removed and analyzed by ELISA after 48 hrs of co-culture. ELISA data is representative of 3 separate blood and liver donors.

As hepatocytes are the primary site of HCV infection and the establishment of persistent HCV infection is associated with a weak CD4^+^ antiviral response, we investigated the possibility that HCV-infected hepatocytes directly modulate CD4^+^ T cell responsiveness. To this end, we established a CD4^+^ T cell/hepatocyte co-culture using a human hepatoma cell line (Huh7.5), stably transfected with the full HCV genome (Huh7.5-FL) or a subgenomic region of HCV (Huh7.5-SG). To examine CD4^+^ T cell function, we assessed the production of the key antiviral Th1 cytokine, IFN-γ. Huh7.5 co-culture resulted in an increase in IFN-γ by CD4^+^ T cells as compared to no hepatocyte treatment. IFN-γ production can be attributed to the CD4^+^ T cells, as no IFN-γ was produced by the hepatocytes. Interestingly, activated CD4^+^ T cells produced less IFN-γ when co-cultured with Huh7.5-FL as compared with Huh7.5 or Huh7.5-SG (Fig. 1A). Despite blood donor variability, IFN-γ suppression was statistically significant (Fig. 1B). CD4^+^ T cells co-cultured with HCV-infected primary hepatocytes also demonstrated decreased IFN-γ production as compared to co-culture with hepatocytes exposed to control serum (Fig. 1C). These results suggest that complete HCV genomic expression within hepatocytes has an immunoregulatory effect on CD4^+^ T cell function.

### HCV^+^ hepatocytes increased the Treg population within the co-cultured CD4^+^ T cells

**Figure 2:**
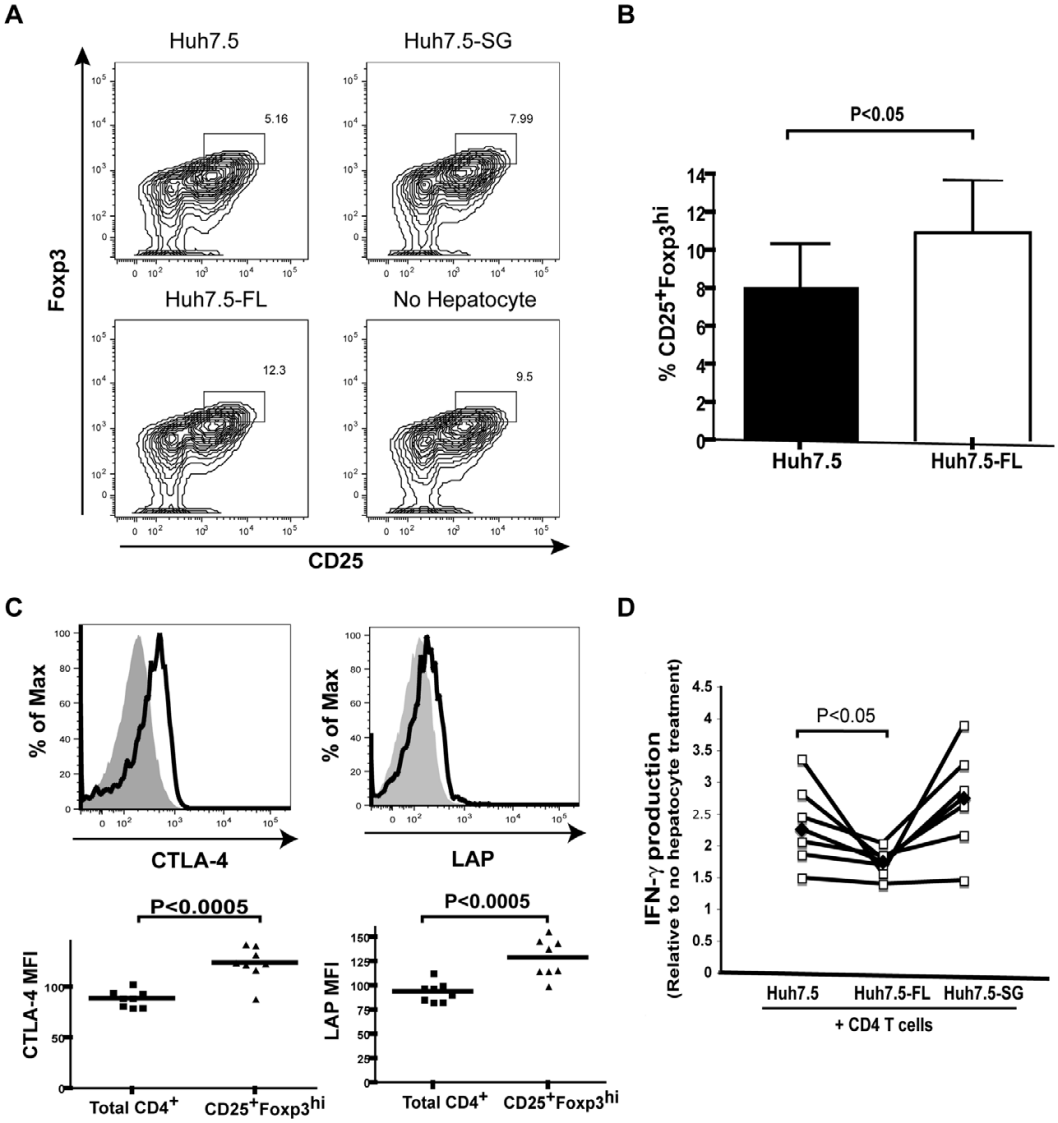
Huh7.5-FL contact results in an increased abundance of regulatory T cells by phenotype. A) Representative flow cytometry analysis of CD25 and Foxp3 staining following CD4^+^ T cell/hepatocyte co-culture. Rectangles show double positive gating and numbers reflect percentage of cells in that gate. B) CD25^+^Foxp3^hi^ data was compiled from 8 healthy CD4^+^ T cell donors. C) Expression of CTLA-4 and LAP in the total CD4^+^ T cell and CD25^+^Foxp3^hi^ populations was assessed from CD4^+^ T cells co-cultured with Huh7.5-FL. Data is presented in histogram with total CD4^+^ T cells represent in solid grey and CD25^+^Foxp3^hi^ cells as the black line. Compiled mean fluorescence intensity (MFI) is shown from experiments with 8 CD4^+^ T cell donors. D) CD4^+^ T cell/hepatocyte co-culture was conducted using CD25-depleted CD4^+^ T cells. IFN-γ production is presented relative to no hepatocyte control. Data are compiled from 7 CD4^+^ T cell donors.

Human CD4^+^ T cells have been found to maintain plasticity after activation. Recent studies support the possibility that after the initial activation of naïve CD4^+^ T cells in the lymph nodes, CD4^+^ T cells are skewed away from a productive antiviral Th1 phenotype [21] and toward a regulatory phenotype in the liver. Regulatory CD4^+^ T cell development can occur as late as 72 hours after TCR engagement [22]. Therefore, the decreased IFN-γ production by CD4^+^ T cells co-cultured with Huh7.5-FL may result from the development of Tregs. Treg development was assessed by the expression of CD25 and Foxp3 in CD4^+^ T cells co-cultured with Huh7.5, Huh7.5-SG or Huh7.5-FL. As shown in Fig. 2A and Fig. 2B, the intracellular CD25 and Foxp3 staining demonstrated a significant increase in CD25^+^Foxp3^hi^ populations among CD4^+^ T cells co-cultured with Huh7.5-FL. Since Foxp3 expression in humans appears to be associated with activation in addition to a regulatory phenotype, we also examined the expression of T cell activation marker, CD69, to determine if Huh7.5-FL merely modulated T cell activation status. CD69 was not upregulated on T cells co-cultured with Huh7.5-FL in comparison to Huh7.5. Rather, CD69 expression decreased with Huh7.5-FL (data not shown). CTLA-4 and LAP (a TGF-β binding protein) have been reported to serve as additional markers for functional CD4^+^ Tregs [23], [24]. Further characterization of the CD25^+^Foxp3^hi^ population supported the notion that these were regulatory cells as they also up-regulated both CTLA-4 and LAP (Fig. 2C).

It is possible that co-culture with Huh7.5-FL enhanced the expansion of naturally occurring regulatory cells present in the CD4^+^ T cell population rather than inducing the development of Tregs. To address this possibility, CD25^+^ cells were depleted from the CD4^+^ T cell population prior to activation, eliminating this pre-existing Treg population. The IFN-γ reduction demonstrated with Huh7.5-FL co-culture was maintained when CD25^+^ cells were depleted, suggesting that Treg enhancement is due to an induction of a Treg population from CD25^-^CD4^+^ T cells (Fig. 2D).

**Figure 3:**
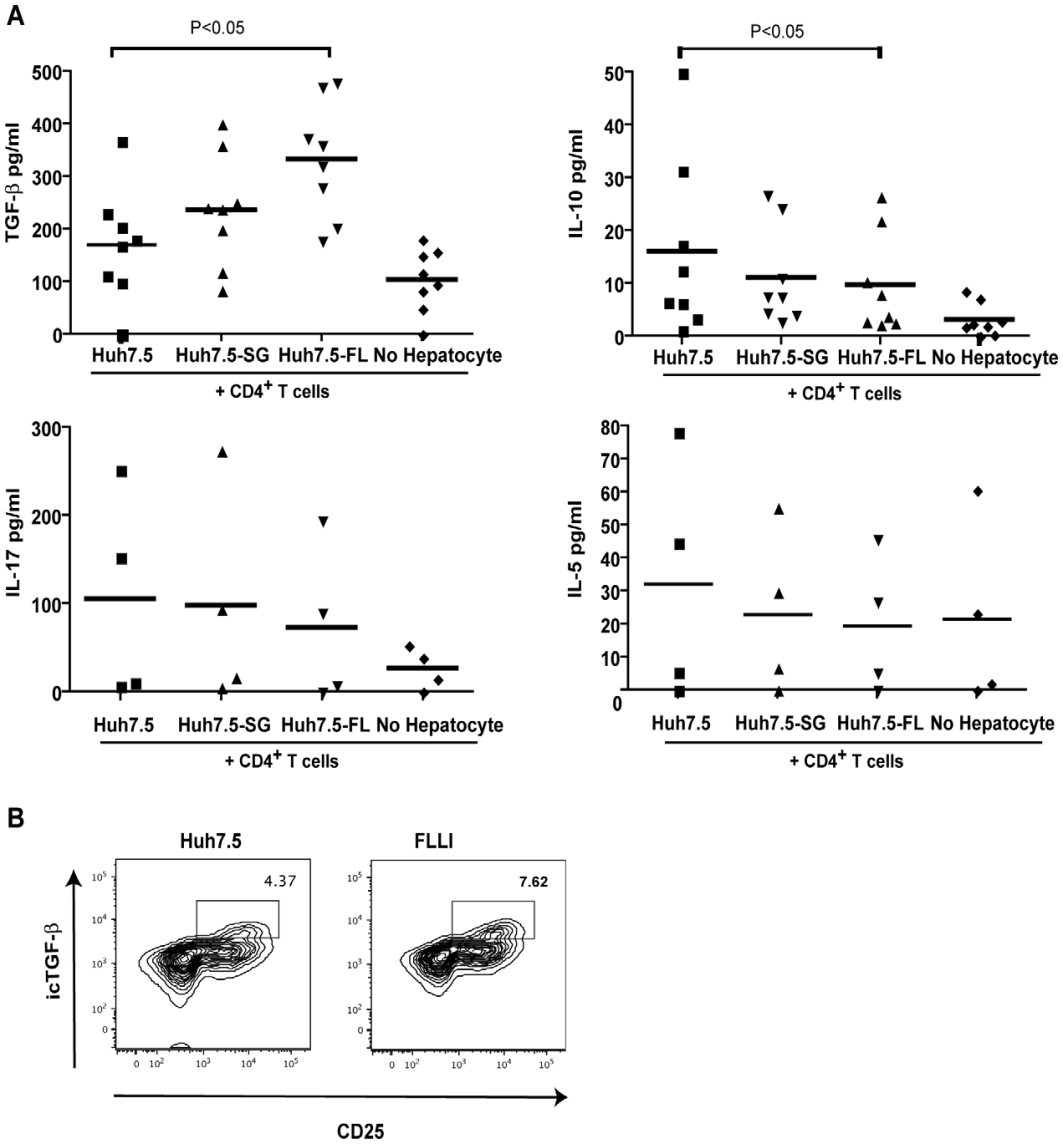
Huh7.5-FL induction of a T regulatory phenotype is associated with an increase in CD4^+^ T cell TGF-β production. A) Cytokines produced by CD4^+^ T cells in hepatocyte co-culture were measured by ELISA (TGF-β and IL-10) or by bead multiplex (IL-17 and IL-5). B) Intracellular TGF-β levels were examined by flow cytometry after hepatocyte co-culture. Gating shows the percentage of TGF-β containing, CD25^+^ T cells. Data are reproducible in 3 independent experiments.

To more fully address phenotypic changes that occurred with hepatocyte/CD4^+^ T cell co-culture, we expanded our studies to examine cytokine production. We found no alteration in Th2 or Th17 cytokines, IL-5 or IL-17. IL-10 production was relatively low and slightly decreased in Huh7.5-FL co-cultures. There was, however, a significant increase in the characteristic Treg cytokine, TGF-β, in Huh7.5-FL co-cultures (Fig. 3A). Intracellular staining confirmed the enhanced TGF-β expression by CD4^+^ T cells in co-culture with Huh7.5-FL (Fig. 3B). These results suggest that the enhanced CD25^+^Foxp3^hi^ cells are inducible Tregs commonly referred to as Th3 cells and characterized by TGF-β production.

### Tregs induced by Huh7.5-FL are capable of suppressing the proliferation of effector T cells

**Figure 4:**
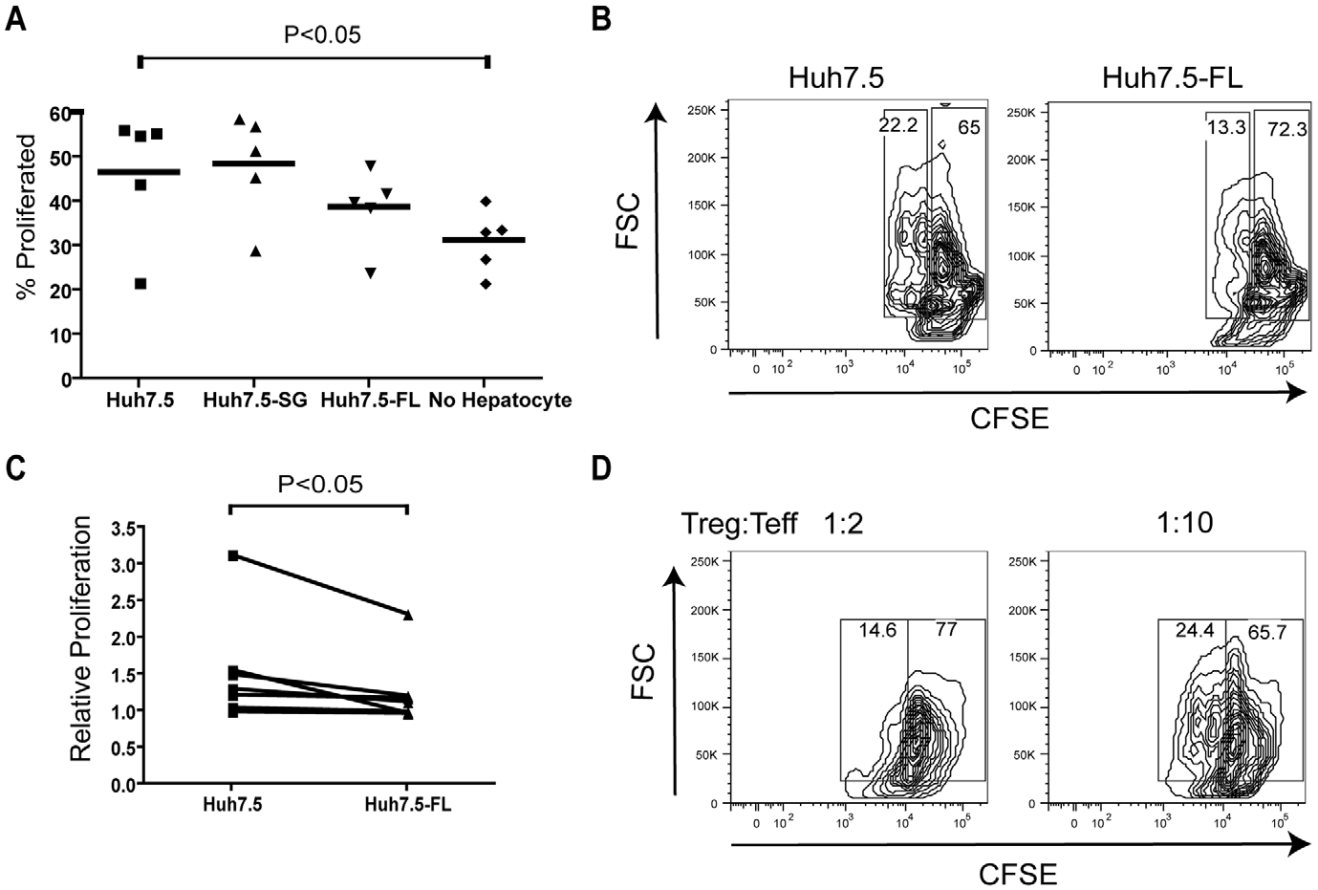
Regulatory T cells developed in Huh7.5-FL co-cultures have suppressive function. A) CFSE-labeled cell CD4^+^ T cells were used in the CD4^+^ T cell/Huh7.5 co-culture in order to examine proliferative response. Cells considered to have divided demonstrate CFSE dilution. Data was compiled from 5 CD4^+^ T cell donors. B, C) Equal numbers of CD4^+^ T cells were removed after co-culture with Huh7.5 or Huh7.5-FL and placed in co-culture with CFSE-labeled CD4^+^ T cells at a ratio of 1∶1 in the presence of plate-bound anti-CD3/CD28. Data were compiled from 7 CD4^+^ T cell donors. D) Isolated CD25^+^ cells from CD4^+^ T cell/Huh7.5-FL co-culture were incubated with CFSE-labeled T effector responder cells. Data are reproducible in 3 independent experiments.

In addition to surface marker expression and production of anti-inflammatory cytokines, Tregs are characterized by the ability to suppress effector T cell proliferation. We first examined the proliferative responses of CD4^+^ T cells in contact with Huh7.5, Huh7.5-SG or Huh7.5-FL by monitoring the dilution of CFSE-labeled CD4^+^ T cells (Fig. 4A). Consistent with published results using other hepatoma lines [19], Huh7.5 enhanced the proliferation of previously TCR-activated CD4^+^ T cells. Although proliferation of CD4^+^ T cells co-cultured with Huh7.5-FL compared to Huh7.5 alone or Huh7.5-SG was not significantly different, there was a trend toward a reduction in proliferation with Huh7.5-FL co-culture.

In order to more directly test the ability of Huh7.5-FL-induced Tregs to suppress proliferation of effector T cells, suppression assays were conducted using autologous CFSE-labeled responder cells. In the suppression assay, activated responder T cells proliferated less when stimulated in the presence of CD4^+^ T cells co-cultured with Huh7.5-FL as compared to Huh7.5 (Fig. 4B). Huh7.5-FL-induced Tregs suppressed effector CD4^+^ T cell proliferation in 7 of the 7 CD4^+^ T cell donors tested (Fig. 4C). The addition of isolated CD25^+^CD4^+^ T cells reduced effector cell proliferation in a dose dependent manner (Fig. 4D). These findings in conjunction with the previous phenotypic changes suggest that there was an enhancement of functional regulatory CD4^+^ T cells within Huh7.5-FL co-cultures.

### TGF-β produced by HCV^+^ hepatocytes contributes to the induction of Tregs

**Figure 5:**
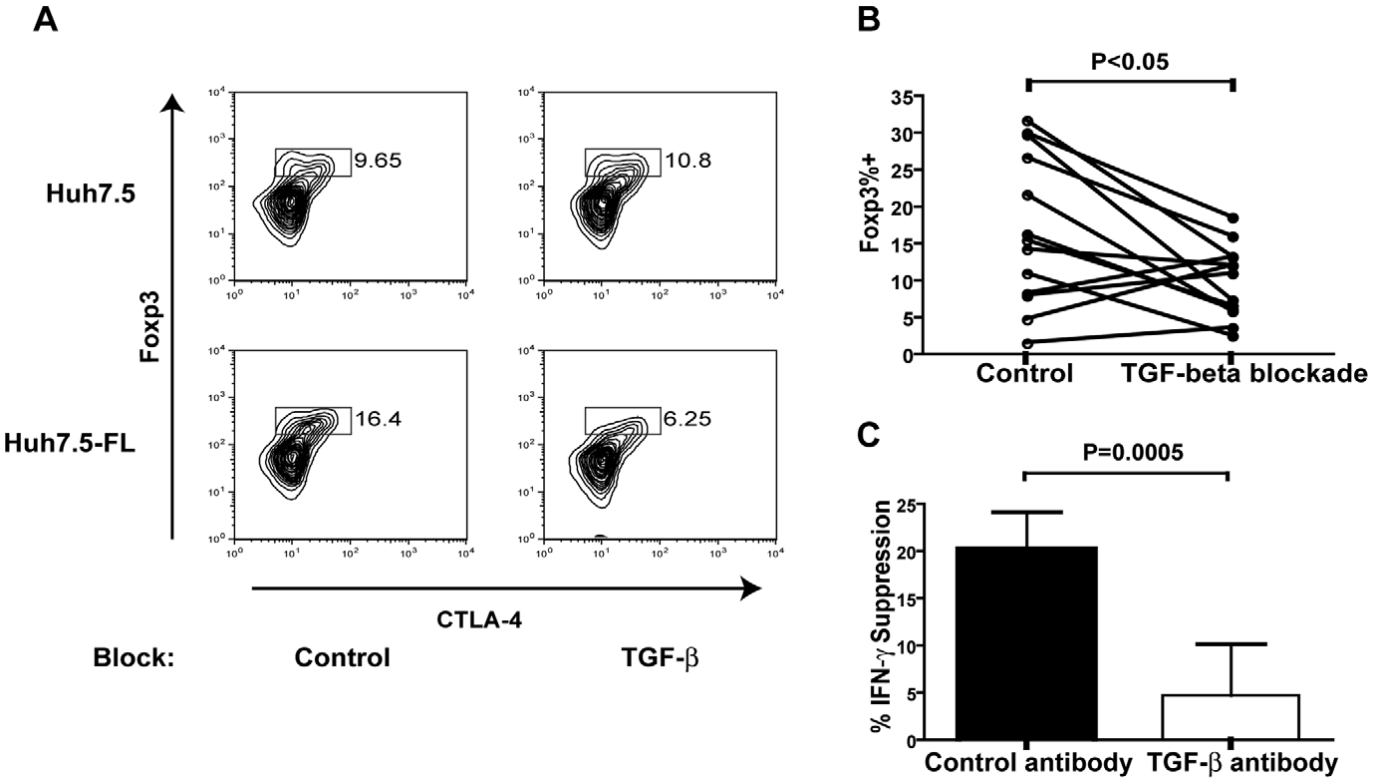
TGF-β contributes to increased regulatory T cell development. TGF-β blocking antibody (0.1 µg/mL) was added to the co-culture in order to monitor impairment of Treg development by (A, B) percentage developing a Treg phenotype or (C) IFN-γ production within the co-cultures. Percent suppression is calculated as ((Huh7.5-Huh7.5-FL)/Huh7.5)*100. Data are compiled from 7 CD4^+^ T cell donors.

TGF-β is known to be a potent inducer of Foxp3 expression and Treg development [9]. Therefore, we examined the ability of a TGF-β blockade to prevent Treg induction in hepatocyte co-culture. As demonstrated in Fig. 5A and 5B, blockade of TGF-β activity resulted in decreased expression of Foxp3. We next evaluated if blockade of TGF-β resulted in recovery of the antiviral response by assessing IFN-γ production in the presence of TGF-β blocking antibody. TGF-β blockade resulted in a partial recovery of IFN-γ production in 6 out of 7 CD4 donors (Fig. 5C).

**Figure 6:**
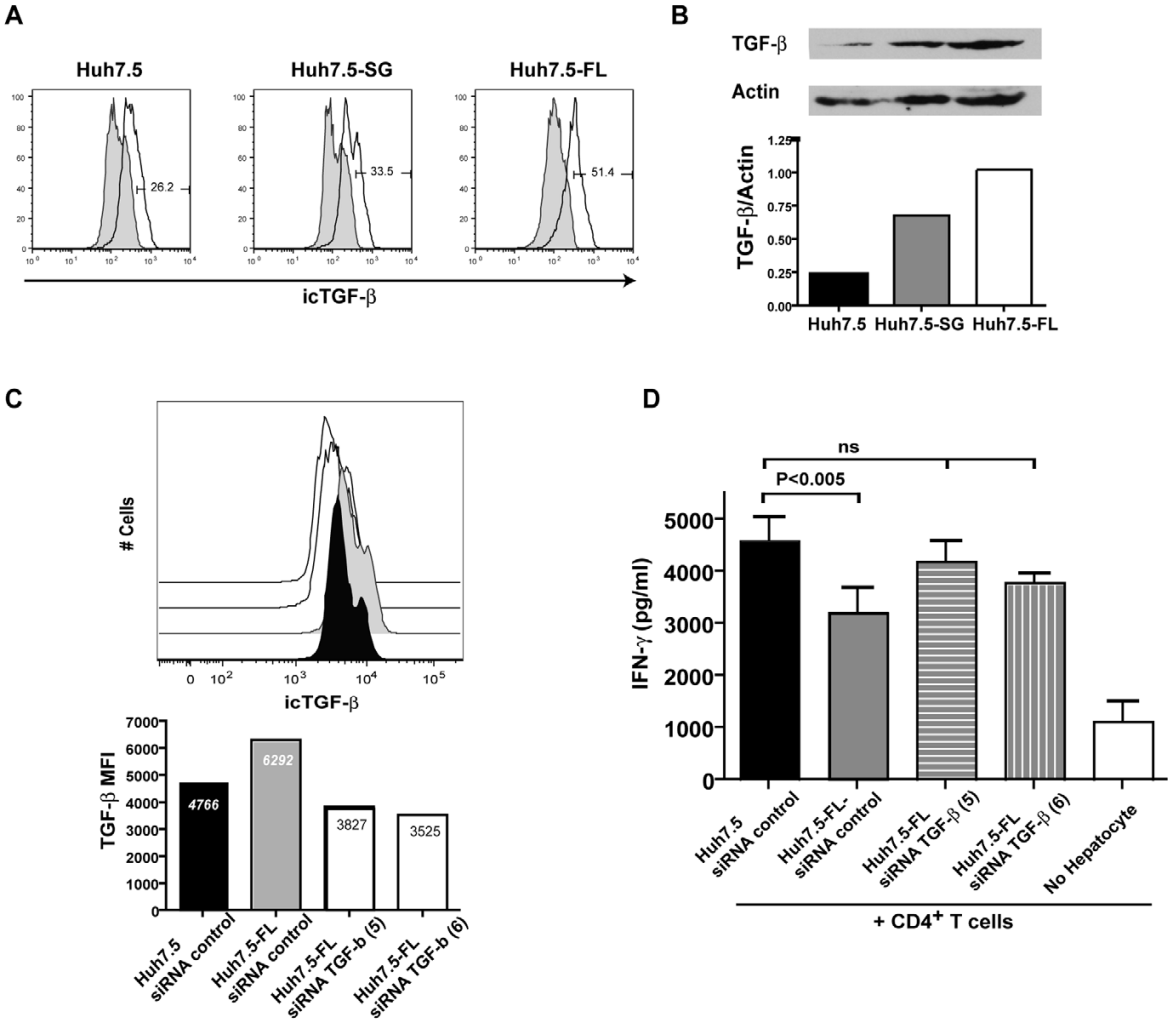
HCV^+^ hepatocytes cause immunosuppression of CD4^+^ T cells due to enhanced TGF-β production. A, B) Hepatocyte TGF-β production was assessed by intracellular flow cytometry (A) and by Western blot (B). Data are reproducible in 3 independent experiments. C, D) TGF-β siRNA knockdown was conducted in hepatocytes using 2 different siRNAs targeting TGF-β and a random RNA sequence not found in the human genome as a control. (C) siRNA knockdown was confirmed by intracellular flow staining of TGF-β. (D) IFN-γ production was assessed by ELISA. Data are compiled from 4 CD4^+^ T cell donors.

Given the crucial role of TGF-β in induction of Tregs and the ability of HCV core protein to induce TGF-β mRNA expression in other hepatocyte cell lines[13], [14], we next determined if the Huh7.5 cell lines were contributing TGF-β to the co-culture. Intracellular TGF-β staining confirmed that Huh7.5-FL produced more TGF-β than did Huh7.5 or Huh7.5-SG (Fig. 6A). This data was also confirmed by western blot analysis (Fig. 6B).

To directly isolate the contribution of hepatic TGF-β production to CD4^+^ T cell immunosuppression, siRNA knockdown of TGF-β was conducted. Two TGF-β targeting siRNA constructs produced a significant reduction in Huh7.5-FL TGF-β production (Fig. 6C). When TGF-β was reduced to the level of Huh7.5 in Huh7.5-FL cells, IFN-γ was present at concentrations comparable to that found in Huh7.5 co-cultures (Fig. 6D). These studies supported the important contribution of hepatic TGF-β to CD4^+^ T cell immunosuppression.

## Discussion

In this report, we demonstrated the contribution of HCV^+^ hepatocytes to regulatory CD4^+^ T cells development. Numerous studies have reported an increased frequency of circulating Tregs during chronic HCV infection [6], [7]. These Tregs are shown to suppress the antiviral activity of CD8^+^ and CD4^+^ T cells and facilitate the establishment of chronic HCV infection. However, the mechanism of Treg induction during HCV infection is unknown. Given the identification of a local TGF-β production as a key determinant of Treg development in mucosal immunity [25], local production of TGF-β in the liver may contribute to the development of Tregs during HCV infection. Indeed, our work supports this possibility by demonstrating a novel mechanism of CD4^+^ Treg development mediated through TGF-β production by HCV^+^ hepatocytes. Other studies support the finding that HCV proteins can induce the production of TGF-β[13] [14] [15]. These findings have clinical relevance as enhanced TGF-β has been identified in HCV infection [26], and appears to influence disease progression as polymorphisms that reduced TGF-β production correlate with HCV clearance[12]. Additionally, a dramatic decrease in TGF-β production is seen in patients that respond to HCV antivirals[27].

This is the first report to show hepatocyte-mediated induction of regulatory T cells to suppress effector cell proliferation and antiviral activities. Hepatocyte-mediated immune regulation appears to require full HCV genome or the structural portion to exert maximal effect. Although partial genomic expression by Huh7.5-SG does exert a partial increase in TGF-β expression, the strongest effect requires the portion of the genome containing the structural proteins. Previous work may suggest that this difference is due to HCV core expression in Huh7.5-FL [13], [14]. Further analysis of genomic expression is necessary in order to define which HCV gene product(s) contribute to the production of TGF-β.

Reduction of TGF-β by antibody or siRNA treatment reveals the important contribution of TGF-β to Treg induction and immunosuppression. Although no virions are produced from Huh7.5-FL cells [28], we cannot discount the possibility that a small amount HCV viral protein may be released and directly interacting with the CD4^+^ T cells. Previous work from our lab demonstrates the ability of HCV core protein to interact with gC1q receptor on the surface of T cells to disrupt TCR signaling and reduce cell proliferation by interfering with early signaling events in naïve T cell activation [29]. Thus, it is unlikely that this mechanism is at work in the hepatocyte/CD4^+^ T cell co-culture as the fully activatedCD4^+^ T cells are exposed to the hepatocyte. Additionally, IFN-γ reduction without a pronounced reduction in proliferation is observed by the CD4^+^ T cells in this co-culture.

Studies of HCV-infected patients have found an increase in HCV-specific Tregs in addition to an increase in Tregs specific for other pathogens such as influenza and tetanus [30]–[31]. Our study, identifying an enhancement of TGF-β production by HCV^+^ hepatocytes, suggests that induction of Tregs of many specificities could occur as they travel through the liver. There may be a relative enhancement of Tregs that react to antigen within the liver due to local sequestration. Although HCV is not considered to be an immunosuppressive virus such as HIV, there is growing evidence that HCV infection predisposes one for a poor response to vaccinations [32], [33] and increased susceptibility to infection with opportunistic pathogens [34], [35]. This could be due in part to the general enhancement of the Treg population.

Hepatic TGF-β production can also account for immunosuppression of other cell types. It is documented that TGF-β can inhibit proliferation and cytokine secretion or can induce a suppressive phenotype in CD8^+^ T cells depending on the strength of the costimulation [36]. This method of CD8^+^ T cell functional suppression is supported by our recent studies that Huh7.5-FL reduced IFN-γ production by activated CD8^+^ T cells (R. Kassel, unpublished). Furthermore, Huh7.5-FL TGF-β production may dampen the immune response by inducing IL-10 production by macrophages [37] or by preventing the maturation of DCs [38]. Further studies are necessary in order to determine the full impact of HCV-infected hepatocytes on regulation of host immunity.

Multiple mechanisms for Treg-mediated suppression of effector T cell functions have been recently reported. Tregs can modulate the activity of T effectors by the production of the immunosuppressive cytokines IL-10 and TGF-β. TGF-β reduces IFN-γ production by activated CD4^+^ T cells and can also induce the expression of IL-10 [39]. This mechanism likely contributed to the reduction in IFN-γ, as the CD4^+^ T cell TGF-β production was substantially enhanced in the Huh7.5-FL co-culture. Tregs can also directly kill effector cells in a cell contact dependent manner. Recent studies report that natural Tregs mediate apoptosis by perforin or granzyme release [40] whereas inducible Tregs require Fas/FasL interaction to induce cell death [41]. Indeed this mechanism could contribute to local immunosuppression as FasL was upregulated and apoptosis enhanced with Huh7.5-FL co-culture (data not shown). Therefore, multiple suppressive pathways may contribute to the reduction in IFN-γ in our system.

The appearance of Tregs generated during this model of HCV infection is contradictory to the development of liver inflammation and cirrhosis during later stages of disease progression. This conflict could be explained by the contribution of other inflammatory cytokines counteracting the TGF-β produced by the infected hepatocytes. One potential cytokine is IL-6 which accumulates in the liver during chronic liver disease [42]. Importantly, high concentrations of IL-6 are able to block TGF-β mediated Treg development and enhance the development of a Th17 population. Enhancement of Th17 cells has been noted in the livers of chronic HCV patients [43]. Additionally TGF-β is known to contribute directly to the induction of fibrosis by stimulating collagen production [44]. A balance of pro- and anti-inflammatory cytokines is likely to contribute for both CD4^+^ T cell phenotype and antiviral activity during the course of HCV infection.

Previous clinical studies of HCV infections have clearly demonstrated the importance of Tregs to the suppression of an effective immune response. Our work establishes the capability of HCV^+^ hepatocytes to induce Tregs from activated CD4^+^ T cells. siRNA data suggests that the hepatocytes are able to affect this change in part by producing the immunosuppressive cytokine, TGF-β. This immunosuppressive influence of HCV^+^ hepatocytes can contribute to a weak immune response and the development of chronic infection.

## Materials and Methods

### Hepatocyte cell line culture and stimulation

Apath LLC (St. Louis, MO) provided parental Huh7.5 cells. Huh7.5 cells harboring full-length (Huh7.5-FL) and subgenomic (Huh7.5-SG) replicons as previously described [28] were a gift from Dr. Charles Rice (Rockefeller University, New York, NY). Huh7.5-FL and Huh7.5-SG were cultured in media containing 750 µg/ml G418 (Invitrogen, Carlsbad, CA) to maintain the viral mRNA. Prior to co-culture, hepatocytes were serum starved for 18 hr, then activated with 0.1 µg/mL rhIFN-gamma (R&D Systems, Minneapolis, MN) for 48 hours.

### Primary hepatocyte culture

Primary hepatocytes (CellzDirect, Pittsboro, NC) were cultured on Matrigel (BD Biosciences, San Jose, CA)-coated six-well plates in Williams E media without phenol red (Sigma-Aldrich, St. Louis, MO) supplemented with L-glutamine, penicillin/streptomycin, HEPES (4-(2-hydroxyethyl)-1-peperazine ethanesulfonic acid) (Gibco BRL, Gaithersburg, MD), hydrocortisone (Sigma-Aldrich), and Insulin Transferrin Selenium (ITS+; BD) at 37°C with 5% CO_2_. Adhesion media was additionally supplemented with 5% vol/vol fetal bovine serum (CellGro; Mediatech, Manassas, VA). Infection of primary hepatocytes was conducted exposing hepatocytes to infectious serum (MOI 0.15–0.05) from HCV-infected patients for 18 hours. HCV serum was provided by Dr. Timothy Pruett. The hepatocytes were washed and incubated for 4 days.

### Isolation and activation of CD4^+^ T cells

PBMC were isolated from buffy coats (Virginia Blood Services, Richmond, VA) (IRB#8233) using Lympholyte-H (Cedarlane Laboratories, Ontario, Canada). All T cell selection was done using CD4 and CD25 positive selection microbeads (Miltenyi Biotech, Bergisch Gladbach, Germany). CD4^+^ T cells were subsequently plated in RPMI supplemented with 1% L-glutamine, 0.1% penicillin/streptomycin, 10% FBS and 10 U/ml rhIL-2 on plates coated with anti-CD3 clone HIT3a (0.5 µg/ml) and anti-CD28 clone CD28.2 (5 µg/ml) (eBioscience, San Diego, CA). CD4^+^ T cells were activated for 72 hrs prior to hepatocyte co-culture.

### Co-culture conditions

Activated hepatocytes were removed from plates by 0.05% trypsin-EDTA (Gibco BRL) and were then plated at 3×10^5^ cells/well in a 24 well plate in DMEM media (+/− mitomycin C at 6.25 µg/ml (Sigma-Aldrich)). Mitomycin was used only in Figures 1A and 1B in order to control for variability of hepatocyte proliferation. However, further experiments were conducted without mitomycin as it had no effect on the outcome of the co-culture. If treated with mitomycin C, hepatocytes adhered to the plate for 90 min and were subsequently washed to remove mitomycin C. Previously activated CD4^+^ T cells were added to the adherent hepatocytes in RPMI media containing rhIL-2 (10 U/ml) at a concentration of 3×10^5^/well. Hepatocyte and CD4^+^ T cell co-cultures proceeded for 48 hrs.

### ELISA cytokine analysis

Supernatants from the co-cultures were collected after 48 hrs and stored at −80° C until analyzed by ELISA for IFN-γ, TGF-β and IL-10 (eBioscience). MIP-1a, IL-1β, IL-5, IL-6, IL-10, IL-17, RANTES and TNF-α were assessed using a cytokine multiplex kit according to manufacturer's instructions (R&D Systems).

### Flow cytometry

CD4^+^ T cells were collected from the co-culture and stained for surface molecules CD4, CD25, CD69, CTLA-4(eBioscience) and LAP (R&D systems). Intracellular staining was performed using cytofix/cytoperm (BD Bioscience) and an anti-FoxP3 antibody (eBioscience). Intracellular TGF-β (IQ Products, Netherlands) was stained similarly, with the addition of Golgistop (BD Bioscience) pre-treatment for 3–6 hrs. A cytokine not expressed by hepatocytes, IL-12 (eBioscience), was used as a negative control. Proliferation assays were performed by staining activated T cells with CellTrace CFSE cell proliferation dye (2.5 µM) (Invitrogen). Flow cytometry was performed using FACS Calibur, FACSCanto (BD Bioscience) and Flowjo software (Treestar inc, Ashland, OR).

### Suppressor function assay

CD4^+^ T cells were magnetically isolated as previously described and labeled with CellTrace CFSE cell proliferation dye. CFSE-labeled CD4^+^ T cells were plated on CD3/CD28 coated plates. CD4^+^ or CD4^+^CD25^+^ selected T cells were added as suppressor cells directly after hepatocyte co-culture. Treg suppressive function was assessed at 72 hrs.

### TGF-β blockade experiments

For blockade of TGF-β, a neutralizing antibody against TGF-β or control antibody was added at a concentration of 0.1 µg/ml (R&D Systems).

### Western blot

Hepatocytes were treated for 48 hrs with 0.1 µg/mL rhIFN-gamma (R&D Systems) prior to cell lysis in lysis buffer (20 mM Tris-HCl pH 7.5, 150 mM NaCl, 1 mM EDTA, 1 mM EGTA, 1% TritonX-100, 2.5 mM pyruvic acid, 1 mM sodium orthovanadate, 1 mM NaF, and Protease Inhibitor Cocktail Set V [Calbiochem]). The lysates were cleared of debris by centrifugation and suspended in 5X sample buffer (125 mMTris-HCl pH 6.8, 10%Glycerol, 2% SDS, 0.00125% Bromophenol Blue [Sigma], 20% β-Me). Samples were then boiled for 5 min and centrifuged for 1 min at 10,000xg. Equal amounts of cell lysate were subjected to SDS-PAGE analysis and transferred to 0.2 µm nitrocellulose paper. Western blot analysis was performed using a rabbit polyclonal Ab against TGF-β (Cell Signaling Technology) or actin-HRP (Santa Cruz Biotechnology, Santa Cruz, CA). Goat anti-rabbit Ig HRP (Cell Signaling Technology) and ECL Plus (Amersham Biosciences, Piscataway, NJ) were used for chemiluminescent detection.

### TGF-β siRNA knockdown

Hepatocytes were plated 24 hrs before the addition of 50 nM siRNA/Lipofectamine 2000 (Invitrogen) complex according to manufacturer's specifications. The siRNA targeting TGF-β (HsTGFB1(5), HsTGFB1(6)) and a negative control siRNA (CtrlAllStars1) were purchased from Qiagen (Germantown, MD). After 72 hrs of siRNA treatment and washing of residual siRNA complexes, activated CD4^+^ T cells were added to siRNA treated hepatocytes at a 1∶1 ratio. TGF-β knockdown was verified using intracellular flow cytometry.

### Statistical Method

All data were analyzed using GraphPad Prism (GraphPad Software, San Diego, CA). Paired and unpaired T tests were used to compare different conditions. P values less than 0.05 were considered significant.
